# Malnutrition, symptom burden and predictive validity of the Patient‐Generated Subjective Global Assessment in Central Australian haemodialysis patients: A cross sectional study

**DOI:** 10.1111/1747-0080.12763

**Published:** 2022-07-29

**Authors:** Lauren Caruana, Liz Nichols, Kelly Lambert

**Affiliations:** ^1^ Cairns Hospital Queensland Australia; ^2^ Department of Nutrition & Dietetics Alice Springs Hospital The Gap Northern Territory Australia; ^3^ University of Wollongong Wollongong New South Wales Australia

**Keywords:** Aboriginal, cross sectional study, haemodialysis, Indigenous, malnutrition, symptom burden

## Abstract

**Aim:**

To (i) describe the prevalence of malnutrition among a cohort of central Australian, predominantly Indigenous, haemodialysis patients and (ii) determine the sensitivity and specificity of the Patient Generated Subjective Global Assessment total score for identification of malnutrition in these patients.

**Methods:**

Cross‐sectional observational study of all patients attending haemodialysis units within the Central Australia Health Service. Patients were assessed using the Patient‐Generated Subjective Global Assessment. Chi‐Square tests were used to determine the association between nutritional status and location, age and gender. Receiver Operator Characteristic curves were used to ascertain the predictive validity for malnutrition of the total score.

**Results:**

Indigenous patients comprised 98% of study haemodialysis patients (*n* = 249/253). One third were male, and 72% were aged between 30 and 59 years. Approximately 29% (74/253) were malnourished, and 93% (69/74) had a total score ≥ 4. The most frequently reported problems that kept malnourished patients from eating were early satiety (32%), no appetite (31%), diarrhoea (26%) and dental problems (24%). Money problems were reported by 32%, as were transport (20%) and depression (19%). The traditional tool cut off score of ≥9 had low sensitivity (50%) for detecting malnutrition. Instead, a score ≥ 3 is suggested due to a higher sensitivity (96%) and specificity (45%).

**Conclusion:**

Malnutrition was found to be common, and we suggest using a Patient‐Generated Subjective Global Assessment total score of ≥3 to improve the identification of malnourished individuals in this cohort of predominantly Indigenous haemodialysis patients. This will significantly increase referrals for dietetic intervention.

## 1 INTRODUCTION

Haemodialysis is one form of renal replacement therapy for those who experience kidney failure. In the most recent census of haemodialysis in Australia (2020), there were approximately 10 916 people undertaking in‐centre haemodialysis.^1^ However, the burden of haemodialysis is not distributed evenly across the Australian population, and Indigenous people comprise 19% of the total Australian haemodialysis population. These differences are amplified when examined according to the level of geographical remoteness,^2^ and gender, with Indigenous females who live in remote areas dominating the dialysis population.^3^

Few studies in Australia have examined the nutritional status of Indigenous Australians who undertake dialysis. Of the single published study of 25 South Australian Aboriginal and 51 non‐Aboriginal South Australian haemodialysis patients in two satellite units, it was found that 35% of Aboriginal patients were malnourished.^4^ The mean Patient‐Generated Subjective Global Assessment (PG‐SGA) score was not significantly different from non‐Aboriginal patients (4.2 ± 3.6 compared with 4.5 ± 3.4). Unfortunately, the authors did not describe the symptom burden of the patients so it remains unknown what factors may be contributing to the high rate of malnutrition documented.

There are also few studies that have examined the predictive validity of the PG‐SGA in the Australian dialysis population, or more specifically the Australian Indigenous dialysis population. Campbell et al.^5^ undertook a prospective observational study of 213 individuals from a single metropolitan in‐centre haemodialysis unit. The prevalence of malnutrition was estimated to be 23.5% and the median PG‐SGA symptom score among the malnourished patients was 3.5 (interquartile range 1.8–7). The most common symptom reported in those who were malnourished was no appetite (60%) and early satiety (36%). A PG‐SGA nutrition impact score ≥ 2 was the strongest predictor of mortality with an area under the curve of 0.86 (95% CI: 0.79–0.93) and sensitivity of 76%. To our knowledge, no additional studies have been published in the Australian context.

Given these evidence gaps, and the important health and financial implications of malnutrition among dialysis patients,^6^ the aims of this study were to: (i) describe the prevalence of malnutrition among haemodialysis patients in the Central Australia Health Service, a patient population dominated by Indigenous Australians and (ii) determine the sensitivity and specificity of the PG‐SGA total score for identification of malnutrition among a cohort of central Australian, predominantly Indigenous, haemodialysis patients.

## 2 METHODS

All maintenance haemodialysis patients attending the acute or satellite haemodialysis units in the Central Australia Health Service were approached to participate in the study. Exclusion criteria included patients with known cognitive impairment, or inability of the carer to assist with completion of the PG‐SGA; or unavailability of an interpreter/Cultural Liaison Officer to assist with communication and completion of the assessment.

Ethical approval was obtained from the Central Australia Human Research Ethics Committee (Approval number:14–264). Patients were approached by either a dietitian or student dietitian and asked to participate in the study, which included a full nutritional assessment completed by the dietitian, and a physical exam using the PG‐SGA tool. The study was conducted in each unit over the course of 1 week in October 2016. Verbal informed consent was obtained from all participants or their guardian.

Dietitians completed the PG‐SGA alongside patients. To ensure consistency between assessors, three team training sessions were conducted prior to undertaking the audit. In addition, all assessments were completed in pairs (with a more senior staff member paired with a less experienced staff member) to ensure accuracy of assessments. A script was also provided to dietitians to ensure questioning was consistent during the assessment.

Section three of the PG‐SGA (i.e. ‘symptoms that have kept me from eating enough during the past 2 weeks’) included one additional prompt. This prompt was used when exploring ‘other’ factors impacting intake and explored if transport impacted access to food. This is widely known to impact the nutritional status in this population group.^7^, ^8^ All patients who were identified as malnourished or at risk of malnutrition, that is, with a PG‐SGA total score ≥ 4 or Subjective Global Assessment (SGA) category of B or C, were triaged and referred to the renal dietitian attached to the haemodialysis unit for follow up.^9^ Those with a PG‐SGA total score of ≥9 or SGA = C, were triaged as a high priority based on known criteria.^10^, ^11^

Demographic and anthropometric data including dry weight, height, and weight history were obtained from the medical records by the renal dietitian. Where dry weight did not correspond to the weight history recorded on the dialysis charts, clarification was sought from the nephrology team or discussion was held between the renal dietitians known to the patient and the Nurse Unit Manager to determine a more appropriate dry weight to record. Responses from patients regarding the presence of wounds was cross checked with medical record files and data on patients under the care of the wound management team. Ethnicity as reported in the medical file was recorded. Dialysis locations were recorded as one of five dialysis units located within the Alice Springs region. Age was retrieved from the medical record, and for the purposes of this study were categorised as 18–29; 30–59; 60–64; 65–74 and ≥75 years. Self‐reported residence was noted and verified by assessors by cross checking against the renal transport bus list (where 95% of patients rely on the bus to get to dialysis). BMI was categorised according to the recommendations from the KDOQI 2020 Clinical Practice Guidelines for Nutrition in Chronic Kidney Disease (CKD).^6^

All statistical analysis was conducted using SPSS software (version 25.0, SPSS Inc., Chicago). Normality of continuous data was determined using the Shapiro–Wilk test. Descriptive data is reported as median and interquartile range or number and proportions. Chi Square tests were used to explore the association between malnutrition status and categorical variables such as gender, place of residence, and dialysis location. The Kruskal–Wallis test or Mann–Whitney *U* was used to explore the relationship between total PG‐SGA score, anthropometric variables (e.g. height, weight) and nutritional status. To ascertain the sensitivity and specificity, and optimal PG‐SGA total cut off score for identifying malnourished haemodialysis patients, the receiver operating characteristics (ROC) curve was used. An area under the curve (AUC) of 0.9–1 indicates the PG‐SGA score was an excellent measure for detecting malnutrition; 0.8–0.89 a good test; and 0.70–0.79 a fair test.^12^ Statistical significance was set at a *p* value of 0.05. Reporting of this study is consistent with the STROBE statement for cross sectional studies.^13^

## 3 RESULTS

Over the study period, a total of 253 patients consented to participate in the study (equating to a response rate of 76%). The study sample was around one third male (35%, 89/253, Table 1), with 98% of the recruited participants identifying as Indigenous. Almost three quarters (72%, 183/253) of the sample were aged between 30 and 59 years old; and one quarter (26%, 65/253) were aged ≥60 years. Approximately one third of the sample resided in either hostel accommodation (28%, 70/253, Table 1) or a private residence (36%, 90/253). Approximately one quarter lived in a town camp (27%, 69/253). One third of the sample had a BMI in the healthy range (37%, 93/253, Table 1). Approximately 15% of the sample had a pressure sore or open wound.

**TABLE 1:** Clinical and demographic characteristics of participants (*n* = 253)

	Total (*n* = 253) number (%)	Well nourished (*n* = 179)	Malnourished (*n* = 74)	*p* value
Male gender	89 (35)	62 (35)	27 (37)	0.78
Age (years)				0.38
18–29	4 (2)	3 (2)	1 (1)	
30–59	183 (72)	135 (75)	48 (65)	
60–64	31 (12)	20 (11)	11 (15)	
65–74	34 (13)	20 (11)	14 (19)	
75 or over	1 (<1)	1 (<1)	0 (0)	
Proportion over 65 years	35 (14)	21 (12)	14 (19)	0.13
Ethnicity				0.89
Caucasian	4 (2)	2 (1.1)	2 (3)	
Indigenous	249 (98)	177 (99)	72 (97)	
Asian	0 (0)	0 (0)	0 (0)	
Other	0 (0)	0 (0)	0 (0)	
Residence^a^				0.38
Hostel	70 (28)	50 (31)	21 (28)	
Town camp	69 (27)	48(27)	21 (28)	
Private Residence	90 (36)	66 (37)	24 (32)	
Other	23 (9)	15 (8)	8 (11)	
Height, m (range)	1.65 (1.59–1.72)	1.65 (1.59–1.72)	1.65 (1.59–1.7)	0.90
Weight, kg (range)	70.5 (60.5–82.0)	72.5 (64.5–86.0)	60.5 (56.0–77.0)	<0.0001
Body mass index (kg/m^2^)				
Underweight BMI: <18.5	14 (6)	6 (3.4)	8 (11)	<0.0001
Healthy weight BMI: 18.5–24.9	93 (37)	53 (30)	40 (54)	
Overweight BMI: 25–29.9	87 (34)	69 (39)	18 (24)	
Obese BMI: ≥30	59 (23)	51 (29)	8 (11)	
Weight loss in previous 6 months				
0–1.9%	225 (89)	165 (92)	60 (81)	0.008
2–5.9%	11 (4)	8 (5)	3 (4)	
6–9.9%	16 (6)	6 (3)	10 (14)	
10–19.9%	1 (<1)	0 (0)	1 (1)	
≥20%	0 (0)	0 (0)	0 (0)	–
Comorbidities				
Cancer	3 (1)	2 (1)	1 (1)	0.88
AIDS	0 (0)	0 (0)	0 (0)	–
Cachexia^b^	0 (0)	0 (0)	0 (0)	–
Trauma	3 (1)	0 (0)	3 (4)	0.02
Pressure sore or open wound	39 (15)	20 (11)	19 (26)	0.004
Fever	2 (<1)	1 (<1)	1 (1)	0.52
Steroids	0 (0)	0 (0)	0 (0)	–

^a^ Definitions or description of residence: hostel; transient short term accommodation for people from remote areas who need to access health services or education, and residents receive three meals per day; Town camp: community living areas that are Aboriginal communities within the town of Alice springs; Private residence: Other: Nursing home or private care organisation.

^b^ Cachexia refers to Pulmonary or cardiac cachexia. Height and weight reported as median and interquartile range. Ethnicity is reported as the ethnicity recorded in the medical file. BMI range categorisation according to Ikizler et al.^6^

The overall prevalence of malnutrition for the Central Australia Health Service haemodialysis population was 29% (74/253, Table 2). Based on PG‐SGA Global Assessment categories, 29% (72/253) were classified as mild/moderately malnourished, and <1% (2/253) were classified as severely malnourished (PG‐SGA category C). The prevalence of malnutrition did not differ according to age category (*p* = 0.38), gender (*p* = 0.78), dialysis location (*p* = 0.08, data not shown) or a patient's place of residence (*p* = 0.38). There was also no significant difference in malnutrition rate between the Indigenous and non‐Indigenous participants (*p* = 0.62).

**TABLE 2:** Malnutrition and nutrition impact symptoms (*n* = 253)

	Total (*n* = 253) number (%)	Well nourished (*n* = 179)	Malnourished (*n* = 74)	*p* value
PG‐ SGA score	4 (2–7)	3 (2–4)	8.5 (6–10)	<0.0001
PG‐SGA category				–
A—well nourished	179 (71)	179	–	
B—mild/mod malnourished	72 (29)	–	72	
C—severely malnourished	2 (<1)	–	2	
Nutrition impact symptoms				–
Constipation *n* (%)	12 (5)	6 (3)	6 (8)	0.11
Dental problems, *n* (%)	32 (13)	14 (8)	18 (24)	<0.0001
Depression, *n* (%)	26 (10)	12 (7)	14 (19)	0.004
Diarrhoea, *n* (%)	31 (12)	12 (7)	19 (26)	<0.0001
Dry mouth, *n* (%)	12 (5)	7 (4)	5 (7)	0.33
Early satiety, *n* (%)	44 (17)	20 (11)	24 (32)	<0.0001
Money problems, *n* (%)	58 (23)	34 (19)	24 (32)	0.02
Mouth sores, *n* (%)	4 (2)	4 (2)	0 (0)	0.20
Nausea, *n* (%)	25 (10)	11 (6)	14 (19)	0.002
No appetite, *n* (%)	32 (13)	9 (5)	23 (31)	<0.0001
Pain, *n* (%)	17 (7)	11 (6)	6 (8)	0.57
Swallowing issues, *n* (%)	9 (4)	2 (1)	7 (10)	0.001
Smells bother me, *n* (%)	14 (6)	5 (3)	9 (12)	0.003
Taste changes, *n* (%)	21 (8)	9 (5)	12 (16)	0.003
Vomiting, *n* (%)	21 (8)	8 (5)	13 (18)	0.001
Transport issues, *n* (%)	28 (11)	13 (7)	15 (20)	0.003
Total PG‐SGA score ≥ 4, *n* (%)	132 (52)	63 (35)	69 (93)	<0.001

There were significant differences in the total PG‐SGA numerical score between nutritional status categories (Table 2). Those who were malnourished had a median total score of 8.5 (IQR: 6–10); while those who were well nourished had a median total score of 3 (IQR: 2–4, *p* < 0.0001). The most frequently reported problem that kept malnourished haemodialysis patients from eating enough were: money problems (Table 2, 32%, *n* = 24/74); early satiety (32%, *n* = 24/74); dental problems (24%, *n* = 18/74); diarrhoea (26%, *n* = 19/74); and no appetite (31%, *n* = 23/74). Transport was reported as a problem impacting the ability to eat enough by 20% (15/74) of malnourished haemodialysis patients. The proportion of patients overall with a total score ≥ 4 indicating that referral to a dietitian was required was 52% (132/253).

When examining symptoms that impacted eating in the prior 2 weeks, there were many important differences between malnourished and well‐nourished groups. This included a higher proportion of dental problems (24%, *p* < 0.0001); depression (19%, *p* = 0.004); diarrhoea (26%, *p* < 0.0001); early satiety (32%, *p* < 0.0001); money problems (32%, *p* = 0.02); nausea (19%, *p* = 0.002); no appetite (31%, *p* < 0.0001); swallowing problems (10%, *p* = 0.001); smells bothering them (12%, *p* = 0.003); taste changes (16%, *p* = 0.003); vomiting (18%, *p* = 0.001); transport issues (20%, *p* = 0.003); and a higher proportion with a total PG‐SGA score of ≥4 (93%, *p* < 0.001) among those who were malnourished.

**FIGURE 1:**
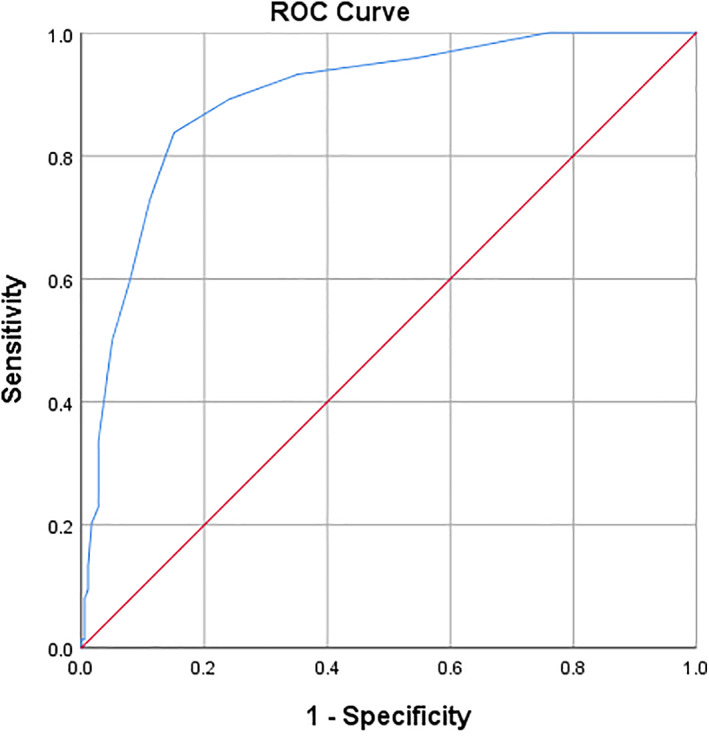
Receiver operating characteristics (ROC) curve plot of the sensitivity and specificity of the nutrition impact score component of the Patient‐Generated Subjective Global Assessment for predicting malnutrition. The area under the curve of 0.894 (95% CI: 0.851–0.936) indicates a good test.

The ROC analysis is shown in Figure 1 and indicated the AUC was 0.894 (95% CI: 0.851–0.936), indicating that the total PG‐SGA score was a good test for malnutrition. In this population group of predominantly Indigenous haemodialysis patients, a PG‐SGA total cut off score of ≥9 as per the author of the tool^14^ only yielded a sensitivity of 50% and specificity of 95%. The sensitivity increased to 96% and specificity of 45% when the total PG‐SGA score was lowered to ≥3; and a sensitivity of 100% and specificity of 24% when lowered to a PG‐SGA total score ≥ 2. A total score cut off ≥3 was therefore considered the most appropriate score to indicate malnutrition and a critical need for intervention in this cohort of haemodialysis patients. The ROC analysis was repeated with non‐Indigenous patients excluded and using boxes 1–4 of the PG‐SGA. This did not substantially change the AUC (AUC 0.893; 95% CI: 0.85–0.936), nor alter the cut off score (sensitivity 96% and specificity 54% for score ≥ 2.5) when examining Indigenous patients only. In contrast, the AUC for boxes 1–4 of the PG‐SGA was 0.804 (95% CI: 0.743–0.868), indicating it was a good‐fair test and not superior to the total PG‐SGA score. Table 3 provides coordinates for the ROC analysis for the total PG‐SGA score (*n* = 253).

**TABLE 3:** Coordinates of the receiver operating characteristics (ROC) curve for total Patient‐Generated Subjective Global Assessment score

Score	Sensitivity	1 Specificity	Specificity
0.0	1.000	1.000	0.00
2.0	1.000	0.760	0.240
3.0	0.959	0.547	0.453
4.0	0.932	0.352	0.648
5.0	0.892	0.240	0.760
6.0	0.838	0.151	0.849
7.0	0.730	0.112	0.888
8.0	0.595	0.078	0.922
9.0	0.500	0.050	0.950
10.0	0.338	0.028	0.972
11.0	0.230	0.028	0.972
12.0	0.203	0.017	0.983
13.0	0.135	0.011	0.989
14.0	0.122	0.011	0.989
15.0	0.095	0.011	0.989
17.0	0.081	0.006	0.994
18.0	0.068	0.006	1.00
19.0	0.041	0.006	1.00

## 4 DISCUSSION

This study provides a comprehensive overview of the nutritional status of a large cohort of predominantly Indigenous haemodialysis patients in the Central Australia Health Service. This represented nearly one fifth (16%) of the national Indigenous haemodialysis population in 2016.^15^ The key findings were that almost one third (29%) of patients were malnourished and symptoms such as anorexia, early satiety, and diarrhoea were more prevalent in malnourished patients. In addition, dental problems and lack of money were also common in malnourished patients. The other key finding of this study was that a PG‐SGA total score of ≥3 appears to be the optimal cut off score for identification of malnutrition in this cohort of predominantly Indigenous haemodialysis patients.

The rates of malnutrition in this study are comparable to those previously documented in Australian dialysis populations. A meta‐analysis of the global prevalence of protein energy wasting estimated 17.9% of the Australian dialysis population were malnourished.^16^ Given that almost all patients in the present study were Indigenous, this increased rate of malnutrition of 29% may indirectly reflect the higher rates of disadvantage and lower socioeconomic status of Indigenous people with kidney disease.^17^ Our findings also extend on previous research, by providing insight into the nutritional status of a remote dialysis population, with prior studies on the prevalence of malnutrition in Australia conducted in urban metropolitan units.^4^, ^5^, ^11^, ^18^ The implications of these findings are concerning, as renal dietitian staffing has repeatedly been shown to be insufficient, both in Australia and abroad.^19^, ^20^, ^21^, ^22^ Given the level of remoteness, and challenges accessing Indigenous health care interpreters^23^, ^24^, ^25^ which are necessary for providing culturally safe care, it is possible that many haemodialysis patients are not receiving dietetic input when most needed.

The 7‐point Subjective Global Assessment is now recommended as the preferred tool for nutrition assessment due to the robust evidence base, particularly for assessment of body composition in dialysis cohorts.^26^ However, the 7‐point SGA does not include an extensive list of symptoms that are known to impact patients with kidney failure. The value of understanding the symptom burden is clearly demonstrated in the present study, with malnourished patients exhibiting an almost three times greater symptom score than well‐nourished patients. The PG‐SGA symptom component thus serves as a useful way to help triage patients in a limited resource environment. Notably, though in the present study, lack of transport and money problems were among the most prevalent factors identified. This suggests that addressing malnutrition in this population requires multifaceted cross‐sectorial approaches that address both the social determinants of health to improve access to affordable food, as well as treatment of symptoms and underlying potentially medical issues that limit intake.

Strategies to address malnutrition in the Indigenous dialysis population are urgently needed. This includes strategies to identify those at risk. Development of a culturally appropriate, specific malnutrition assessment tool for Indigenous Australians may be useful. Recent work on malnutrition in the central Australian inpatient setting identified that a mid‐upper arm circumference of <23 cm demonstrated a strong positive predictive value for malnutrition (96% 95% CI: 89–99.2).^27^ The Adult Nutrition Tool,^28^ an adaptation of the Malnutrition Screening Tool^29^ is also routinely used in the inpatient setting at Alice Springs Hospital and could be used in the dialysis setting. Exploration of symptoms clusters would also be beneficial. While, it is well known that the symptom burden is high in dialysis patients,^30^ examination of how these symptoms cluster together and influence malnutrition risk and quality of life is relatively unexplored. An improved understanding of symptom clusters may assist with treatment priorities and contribute to an improved quality of life.^31^

The critical importance of involving Indigenous consumers in any strategies designed to reduce malnutrition also cannot be understated. A 2016 report identified that more than 75% of all new dialysis patients were required to relocate from a remote community to commence dialysis.^32^ This has been reported in previous studies to create a profound sense of dislocation to be away from family and social ties,^33^ and more importantly disrupted access to traditional foods, and methods for accessing and consuming these foods.^7^ In the present study, approximately one third of participants resided in a hostel (where three meals per day are provided) or another form of temporary accommodation. Despite this, place of residence was not associated with a poorer nutritional status. Future work should explore the nature of food insecurity among Indigenous patients undergoing dialysis, as well as engagement of hostels with consumers regarding meal provision. Potential strategies such as increasing access and availability to traditional foods may be useful. Psychosocial interventions, such as those being tested in the ongoing WICKD trial^34^ may also be informative for helping to address the high levels of depression seen in this study, which will also impact oral intake.

There are several strengths to this work. Researchers assisted patients to complete the PG‐SGA and ensured training among assessors occurred in the week prior to assessment to reduce intra‐assessor variability. Assessors were also dietitians well known to the patient cohort and this may have increased participation and trust among patients. However, there are several limitations. The study was not able to examine relationships between nutritional status and dialysis vintage, dialysis clearances (urea reduction ratios or dialysis adequacy) or any biochemical markers. The findings may not be generalisable to other Australian dialysis units as the proportion of Indigenous patients in this study was significantly higher.

To conclude, this study has found that malnutrition appears to be present in about one in three haemodialysis patients from the Central Australia Health Service. The symptom burden among these patients is also high. Future work should explore symptom clusters to help to develop and triage new models of care, in addition to interventions to assist with financial and social factors impacting nutritional status. In the absence of specific nutritional assessment tools, the authors suggest use of the PG‐SGA using a total score of ≥3 to improve the identification of malnourished individuals in this cohort of predominantly Indigenous haemodialysis patients. This will significantly increase referrals for dietetic intervention.

## AUTHOR CONTRIBUTIONS

LC and LN designed and conducted the research; KL analysed the data; LC, LN and KL wrote and revised the paper. LC and LN had primary responsibility for the final contents. All authors read and approved the final manuscript. The authors acknowledge Margo Bell and Clare Brown for assistance with data collection.

## CONFLICT OF INTEREST

The authors declare no conflicts of interest.

## Data Availability

The data that support the findings of this study are available from the corresponding author upon reasonable request.
